# Intracystic Glucose Measurement for On-Site Differentiation Between Mucinous and Non-Mucinous Pancreatic Cystic Lesions

**DOI:** 10.3390/cancers16244198

**Published:** 2024-12-17

**Authors:** Angelo Bruni, Leonardo Henry Eusebi, Andrea Lisotti, Claudio Ricci, Marcello Maida, Pietro Fusaroli, Giovanni Barbara, Riadh Sadik, Nico Pagano, Per Hedenström, Giovanni Marasco

**Affiliations:** 1 IRCCS Azienda Ospedaliero Universitaria di Bologna, Policlinico S. Orsola, 40138 Bologna, Italy; angelo.bruni4@unibo.it (A.B.); claudio.ricci6@unibo.it (C.R.); giovanni.barbara@unibo.it (G.B.); 2 Department of Medical and Surgical Sciences, University of Bologna, 40138 Bologna, Italy; 3 Gastroenterology Unit, Hospital of Imola, University of Bologna, 40138 Bologna, Italy; a.lisotti@ausl.imola.bo.it (A.L.); pietro.fusaroli@unibo.it (P.F.); 4 Department of Medicine and Surgery, University of Enna ‘Kore’, 94100 Enna, Italy; marcello.maida@unikore.it; 5 Gastroenterology Unit, Umberto I Hospital, 94100 Enna, Italy; 6 Department of Gastroenterology and Hepatology, Sahlgrenska University Hospital, Gothenburg University, 413 45 Gothenburg, Sweden; riadh.sadik@gu.se (R.S.);; 7 Gastroenterology Unit, Department of Oncological and Specialty Medicine, University Hospital Maggiore della Carità, 28100 Novara, Italy

**Keywords:** EUS–FNA, pancreatic cyst, glucose, CEA, mucinous cyst

## Abstract

Background: Pancreatic cystic lesions (PCLs) are frequently detected incidentally and vary from benign to malignant. Accurate differentiation between mucinous (M-PCLs) and non-mucinous PCLs (NM-PCLs) is essential for appropriate management. This study aims to validate the accuracy of on-site glucose measurement using a glucometer with a cut-off of 50 mg/dL for distinguishing M-PCLs from NM-PCLs. Methods: In this prospective multicenter study, conducted at three European academic hospitals, patients who underwent endoscopic ultrasound-guided fine-needle aspiration for PCLs between 2019 and 2020 were included. On-site glucose measurement was performed using a conventional glucometer. Data on demographics, clinical features, EUS findings, and histopathology were collected. Results: Fifty patients were enrolled, with 37 having glucose levels < 50 mg/dL and 13 ≥ 50 mg/dL. M-PCLs were more common in the <50 mg/dL group (81%) compared to the ≥50 mg/dL group (23%, *p* < 0.001). The median CEA was higher in the <50 mg/dL group (146 ng/mL) than in the ≥50 mg/dL group (3 ng/mL, *p* = 0.047). On-site glucose testing < 50 mg/dl demonstrated a sensitivity of 93.2%, a specificity of 76.5%, and an accuracy of 89% for detecting M-PCLs with an AUC of 0.74 and an OR of 14.29 (*p* < 0.001). In comparison, CEA > 192 ng/mL had a sensitivity of 55.6%, a specificity of 87.5%, and an accuracy of 75.8% for M-PCLs, with an AUC of 0.65 and an OR of 4.44. Conclusions: On-site glucose measurement using a glucometer with a cut-off of <50 mg/dL is a highly accurate, rapid, and cost-effective method for differentiating M-PCLs from NM-PCLs. Our results validate the glucose cut-off in a multicentric prospective cohort supporting its integration into standard diagnostic protocols for PCLs.

## Simple Summary

This study investigates a simple and cost-effective method to differentiate between mucinous and non-mucinous pancreatic cystic lesions (PCLs) during EUS–FNA. The proper identification of these lesions is crucial to ensure appropriate treatment. Current methods can be invasive or expensive; therefore, this research aims to validate the accuracy of using a conventional glucometer to measure glucose levels directly from the cyst fluid. The study found that glucose levels below 50 mg/dL were highly accurate in identifying mucinous PCLs. These findings could improve diagnostic protocols, providing a faster and more accessible way to effectively manage PCLs.

## 1. Introduction

Pancreatic cystic lesions (PCLs) are increasingly detected incidentally due to the widespread use of radiological imaging, with a pooled prevalence of 8% in the general population [1]. These lesions vary widely, ranging from benign entities with minimal malignant potential to premalignant and malignant lesions [2,3].

Accurate differentiation between lesions with no malignant potential and those that are premalignant or malignant is essential to prevent the morbidity associated with the overtreatment of benign lesions and to identify patients who require active surveillance or surgical intervention [4,5]. A major challenge in this context is distinguishing between mucinous PCLs (M-PCLs) and non-mucinous PCLs (NM-PCLs), as most premalignant PCLs are mucinous [6,7,8]. However, among non-mucinous lesions, there are cystic lesions, such as cystic neuroendocrine tumors, solid pseudopapillary neoplasms, cystic metastatic epithelial neoplasms, cystic ductal adenocarcinomas, and others, with a high risk of neoplastic transformation [6,9,10,11]. Current guidelines recommend performing endoscopic ultrasound (EUS) for presumed M-PCLs with worrisome features (WF) on cross-sectional imaging [12]. EUS evaluation should include guided fine-needle aspiration (FNA) for cyst fluid analysis, combining cytology with carcinoembryonic antigen (CEA) levels [7,13]. However, cyst fluid cytology has a low sensitivity, and differentiation between M-PCLs and NM-PCLs often relies on CEA levels [14,15]. Despite its use, CEA has shown suboptimal performance for distinguishing M-PCLs, with a sensitivity of 63% and a diagnostic accuracy of 79% at a cut-off value of ≥192 ng/mL [16]. Moreover, the optimal cut-off value for CEA remains debated.

Meanwhile, intracystic glucose has emerged as a promising biomarker for distinguishing between M-PCLs and NM-PCLs. M-PCLs tend to have lower intracystic glucose concentrations, likely due to the higher glucose uptake in premalignant PCLs. Glucose is an inexpensive and widely available biomarker; several studies have shown its superiority over intracystic CEA [17,18].

The possibility of obtaining real-time glucose concentration represents an advantage and enhances diagnostic accuracy and clinical decision making. This method is particularly easy to perform since it requires only a small quantity of pancreatic cyst fluid (PCF), making it feasible even in cases where the fluid volume is limited [19,20,21]. Additionally, the lower cost and wide availability of glucometers further support their use in routine clinical practice, potentially reducing the need for more invasive and expensive diagnostic procedures.

A recent meta-analysis demonstrated that measuring glucose in PCF using a cutoff of <50 mg/dL has a significantly higher sensitivity compared to CEA in differentiating mucinous from non-mucinous pancreatic lesions, with a sensitivity of 91% and a specificity of 86% [22]. However, combining glucose testing with CEA did not improve the diagnostic accuracy compared to glucose testing alone. Moreover, data from the most recent meta-analysis and a trial conducted by Ribeiro et al. in 2024 showed that intracystic glucose measurement with a cut-off of <50 mg/dL has a superior diagnostic performance compared to CEA in distinguishing between the two types of pancreatic cysts; however, the cut-off value has not yet been validated in prospective studies [20,22]. Therefore, the aim of this study is to validate the accuracy of pancreatic intracyst glucose determination using an on-site glucometer, with a cut-off of 50 mg/dL, for differentiating M-PCLs from NM-PCLs in a multicenter prospective cohort.

## 2. Materials and Methods

### 2.1. Population and Study Design

This is a prospective observational multicenter study conducted at University Hospital Sant’Orsola in Bologna (Italy), Hospital of Imola (Italy) and Sahlgrenska University Hospital in Gothenburg (Sweden). Consecutive patients aged 18 years or older who underwent EUS–FNA for a pancreatic cystic lesion between 2019 and 2020, and in whom PCF was obtained, were eligible for inclusion. All patients underwent glucose determination performed using an on-site glucometer. All collected data were entered into an electronic database. Patients’ demographics and clinical features (gender, age, indication for EUS), along with data regarding the EUS features of cysts (location, size, morphology, wall thickness, presence of mural nodules/solid components and main pancreatic duct connection) and histopathological findings were collected from individual electronic clinical records.

### 2.2. Sample Collection

PCF samples were prospectively collected during EUS–FNA procedures. These procedures were performed by specialist endosonographers using standard techniques. Cystic lesions were punctured using 19-gauge or 22-gauge EUS–FNA needles with commercially available needles (Expect Slimline^®^ Boston Scientific, Watertown, MA, USA, EchoTip^®^ Cook Medical, Limerick, Ireland and SonoTip^®^ Mediglobe GmbH, Munich, Germany), according to the endosonographers’ preferences. For patients with more than one cystic lesion, only the largest lesion was considered for analysis. The PCF was obtained by aspirating the fluid directly from the lesion. Uniform training was provided to all operators to ensure consistency in sample handling and testing. On-site glucose measurement was performed using a conventional glucometer (Accu-Check^®^ Infom II, Roche, Rotkreuz, Switzerland), with a range between 10–600 mg/dL. The device was consistently calibrated according to the manufacturer’s instructions to ensure reproducibility and accuracy. All samples with glucose levels < 10 mg/dL were recorded and analyzed with the median value within this range (5 mg/dL). In patients with an adequate volume of cystic fluid, CEA levels were determined.

### 2.3. Outcomes

The primary outcome was the assessment of the diagnostic accuracy of glucose testing, using an on-site glucometer, in PCF for distinguishing between M-PCLs and NM-PCLs using a cut-off of 50 mg/dL. The secondary outcome was the evaluation of the diagnostic performance of CEA > 192 ng/mL in the differential diagnosis of M-PCLs and NM-PCLs.

### 2.4. Gold Standard for Diagnosis

The gold standard for the diagnosis of PCLs was the pathology of the surgical specimen for those who underwent pancreatectomy. Otherwise, a combination of cytology on EUS–FNA samples, cross-sectional imaging (MRI and/or CT), multidisciplinary discussion, including expert pancreatic surgeons, gastroenterologists, radiologists, and pathologists, and at least a 12-month follow-up was used to categorize PCLs as either mucinous (M-PCLs) or non-mucinous (NM-PCLs).

### 2.5. Statistical Analysis

Data are presented as counts and percentages for categorical variables and as median and interquartile ranges (IQRs) for continuous variables. Categorical variables were compared using chi-squared or Fisher’s exact tests, as appropriate, while continuous variables were compared with the Mann–Whitney U test. Differences between glucose < 50 or ≥50 mg/dL and between M-PCLs and NM-PCLs were calculated. Univariate logistic regression analyses were performed to assess the association between glucose levels and CEA with M-PCLs. Results are reported as odds ratio (OR) with a 95% confidence interval (CI). An OR with a 95% CI < 1 indicated that the covariate reduced the risk of M-PCLs and associated clinical features, whereas an OR with a 95% CI > 1 indicated increased risk. An OR with a 95% CI of 1 indicated no significant influence. Multivariate analysis was a priori not performed due to the small sample size. Two-sided probability values with *p*-values < 0.05 were considered statistically significant. Statistical analyses were conducted using SPSS 13 (SPSS Inc., Chicago, IL, USA).

## 3. Results

### 3.1. Demographics

We enrolled a total of 50 patients, of whom, according to intra-cystic glucose levels, 37 (74%) had glucose levels below 50 mg/dL and 13 (26%) had glucose levels equal to or above 50 mg/dL. The gender distribution was similar (*p* = 0.8), with 57% females in the glucose < 50 mg/dL group and 62% females in the glucose ≥ 50 mg/dL group. The median age for the entire cohort was 70 years, with the median age being 71 years for the glucose < 50 mg/dL group and 66 years for the glucose ≥ 50 mg/dL group, without a significant difference (*p* = 0.4). Additionally, there was no statistically significant association between diabetes mellitus (DM) and intra-cystic glucose levels < 50 mg/dL (*p* = 0.15). Demographics and clinical data are detailed in Table 1.

### 3.2. Endoscopic Features

Both groups had a median of 1 cyst without significant numerical differences. The maximum cystic diameter was 20 mm in the glucose < 50 mg/dL group and 35 mm in the glucose ≥ 50 mg/dL group (*p* = 0.017). In both groups, there were 3 patients with mural nodules, showing no significant differences (*p* = 0.2); the maximum diameter and their vascular enhancement was also not significantly different (*p* = 0.7 and *p* = 0.1, respectively). The size of the main pancreatic duct was significantly larger in the glucose < 50 mg/dL group (median 3 mm) compared to the glucose ≥ 50 mg/dL group (median 2.5 mm, *p* = 0.027). The distribution of cysts across different regions of the pancreas did not differ significantly between the groups (*p* = 0.2). Cyst wall thickening was more common in the glucose ≥ 50 mg/dL group (46%) than in the glucose < 50 mg/dL group (14%, *p* = 0.023). Peripancreatic adenopathy was more common in the glucose ≥ 50 mg/dL group (*n* = 3, 23%) compared to none in the glucose < 50 mg/dL group (*p* = 0.015).

### 3.3. Cyst Fluid and Serum Biomarkers

As for cystic CEA levels, the overall median was 41 ng/mL. In the intracyst glucose < 50 mg/dL group, the median CEA value was significantly higher (146 ng/mL) compared to the glucose ≥ 50 mg/dL group (3 ng/mL, *p* = 0.047). As for serum CA19.9, median values were 22 U/mL and 47 U/mL, respectively (*p* = 0.9). The serum amylase levels presented a significant difference; the glucose < 50 mg/dL group had a median of 326 U/L, while the glucose ≥ 50 mg/dL group had a median of 5 U/L (*p* = 0.031). Additionally, a strong positive correlation was observed between on-site and laboratory glucose measurements across 22 paired samples (Pearson’s coefficient: 0.996, *p* < 0.001; Spearman’s coefficient: 0.980, *p* < 0.001), with comparable mean values and standard deviations (52.0 ± 46.77 mg/dL vs. 53.77 ± 48.50 mg/dL). Consequently, intracystic glucose levels from the laboratory evaluation were significantly different among the two groups. The final diagnosis of M-PCLs versus NM-PCLs was significantly different between the groups, with M-PCLs being more common in the glucose < 50 mg/dL group (81% vs. 23%, *p* < 0.001) (Table 2).

### 3.4. Mucinous and Non-Mucinous Pancreatic Cyst Features

When comparing M-PCL and NM-PCL groups, the median age was higher in M-PCLs (71 years) than NM-PCLs (65 years, *p* = 0.017). Glucose levels were significantly lower in the M-PCL group [median 22 mg/dL (14, 29)] compared to the NM-PCL group [median 84 mg/dL (29, 100), *p* < 0.001]. Consequently, intracyst glucose levels with on-site evaluation < 50 mg/dL were significantly different in M-PCLs (91%) compared to NM-PCLs (41%, *p* < 0.001). These results were also confirmed in the subgroup of patients with available intracystic glucose measurements performed in the laboratory. The CEA, CA19.9, and amylase levels did not show significant differences between groups (*p* = 0.10, *p* = 0.078, and *p* = 0.12, respectively). Both groups had a median of 1 cyst, but there was no significant difference in maximum cyst diameter, mural nodule size, or main pancreatic duct size (*p* > 0.05). Gender distribution, diabetes mellitus, cyst location, and the presence of mural nodules were similar (*p* > 0.05). Cyst wall thickening was significantly more common in NM-PCLs (47%) compared to M-PCLs (9.1%, *p* = 0.004). Peripancreatic adenopathy did not differ significantly (*p* = 0.3). Fine-needle aspiration cytology showed significant differences between the groups, with serous cysts more common in NM-PCLs (59%) compared to M-PCLs (6.1%, *p* < 0.001). The FNA cytology results demonstrated a significant difference between M and NM-PCLs (*p* < 0.001). Serous cysts were more commonly associated with NM-PCLs (59%) compared to M-PCLs (6.1%) and high-grade dysplasia was found exclusively in M-PCLs (18%). Additionally, only two samples were non-diagnostic for insufficient specimens, representing 4% of the total. High-grade dysplasia and no neoplastic cells were more common in M-PCLs (18% and 52%, respectively) compared to NM-PCLs (0% and 35%, respectively, *p* < 0.001). The histopathology on surgical specimens did not show significant differences between the groups (*p* = 0.3) (Table 2).

### 3.5. Accuracy of Intracystic Glucose Evaluation for Differentiating M-PCLs vs. NM-PCLs

The ROC curve for intracystic glucose concentration < 50 mg/dL to differentiate between M-PCLs and NM-PCLs yielded an area under the curve (AUC) of 0.74 [95% confidence interval (CI): 0.618 to 0.879]. However, for CEA > 192 ng/mL, the AUC was 0.65 (95% CI: 0.487 to 0.817). The logistic regression analysis revealed that the odds ratio (OR) for glucose levels < 50 mg/dL in predicting M-PCLs versus NM-PCLs was 14.29 (95% CI ranging from 3.09 to 65.99, *p* < 0.001). In comparison, the OR for CEA > 192 ng/mL was lower (4.44, 95% CI ranging from 0.74 to 26.68, *p* = 0.103). Glucose levels < 50 mg/dL had a sensitivity of 93.2%, a specificity of 76.5%, and an overall accuracy of 89% in distinguishing M-PCLs from NM-PCLs, with a positive predictive value (PPV) of 87.5% and a negative predictive value (NPV) of 76.2%. In comparison, CEA levels > 192 ng/mL had lower sensitivity (55.6%) but higher specificity (87.5%), with an overall accuracy of 75.8% (Table 3).

## 4. Discussion

Accurately distinguishing between mucinous and non-mucinous PCLs is crucial for their appropriate surveillance and treatment. Traditional methods, such as morphological imaging features and cytological evaluation, have shown limited accuracy [5]. Several biomarkers have been explored, with intracystic CEA levels being one of the most common [23]. Despite its widespread use, CEA has several limitations as a biomarker for PCLs. Its sensitivity, as seen in our study and others, remains suboptimal, and the debate surrounding the optimal cut-off value further complicates its clinical application [24,25]. The use of glucose levels as an alternative biomarker has shown promise, with several studies indicating its higher accuracy and reproducibility [26,27,28,29].

Our study supports these findings, demonstrating a strong association between low intracystic glucose levels (<50 mg/dL) and mucinous cysts, with an odds ratio of 14.29 (95% CI: 3.09 to 65.99, *p* = 0.0001) and an accuracy of 89%. Moreover, the feasibility of performing on-site glucose analysis by glucometry offers a significant clinical advantage by providing immediate results, thus improving diagnostic accuracy and decision making.

This rapid and cost-effective approach requires significantly lower fluid volumes compared to CEA analysis, making it particularly advantageous in clinical scenarios where the cyst fluid volume is limited. Given the frequent challenge of obtaining sufficient fluid (>1 mL) for traditional CEA testing, the minimal fluid requirement for glucose analysis offers a substantial clinical advantage, ensuring accurate diagnoses even in cases where fluid aspiration is challenging.

Our logistic regression analysis identified that glucose and CEA were significant predictors for mucinous cysts. Among these, glucose showed the highest diagnostic accuracy, consistent with previous studies.

For instance, a study by Park et al., in 2013, demonstrated that glucose levels were significantly lower in mucinous cysts compared to non-mucinous cysts, supporting our findings [30]. A 2021 meta-analysis by McCarty et al. included data from over 500 patients [22]. The analysis demonstrated that intracystic glucose had a pooled sensitivity of 91% and a specificity of 94% for identifying mucinous PCLs, compared to 56% sensitivity and 85% specificity for CEA. Additionally, the overall diagnostic accuracy was significantly higher for intracystic glucose (94%) than for CEA (85%).

Ribeiro et al., in 2024, determined that intracystic glucose measurement with a cut-off of <50 mg/dL showed superior diagnostic performance compared to CEA in distinguishing between mucinous and non-mucinous pancreatic cystic lesions [20]. This study noted that on-site glucose testing provided high sensitivity (93.2%) and specificity (76.5%), and correlated excellently with laboratory measurements, making it an effective and accessible biomarker for PCL characterization.

The potential impact of these findings on clinical practice is substantial. The misclassification of M-PCLs as NM presents a significant clinical risk, potentially leading to the under-management of lesions with malignant potential. Although intracystic glucose analysis demonstrates a high PPV of 87.5%, the risk of false-positive and false-negative results remains, particularly in borderline cases. Similarly, CEA levels, despite their frequent use, exhibit limited sensitivity and specificity, further emphasizing the potential for diagnostic inaccuracies when relying solely on this biomarker.

On-site glucometry can streamline the diagnostic process, allowing for quicker and more accurate differentiation of PCLs, with an accuracy comparable to that of laboratory glucose measurements. This can lead to better patient management, reducing unnecessary surveillance and interventions for non-mucinous cysts while ensuring timely treatment for mucinous cysts. However, the presence of diabetes does not appear to influence concentrations of intracystic glucose.

Our study has some strengths. It is a prospective multicenter study that involves experienced endosonographers and uses different devices for PCL assessment. Although variability between centers may arise due to differences in endosonographer techniques, local sampling procedures, patient conditions, and the volume of fluid aspirated, this diversity also improves the generalizability of our findings across a range of clinical environments.

Despite its strengths, this study has several limitations. The relatively small size of our cohort may impact the generalizability of the results. Moreover, definitive diagnoses were established via cytology in 5 cases, surgical histopathology in 10 cases, and a multidisciplinary consensus approach in 18 cases. Specifically, all cysts classified as mucinous during the multidisciplinary discussions demonstrated clear communication with the main pancreatic duct, as visualized on EUS or magnetic resonance cholangiopancreatography. However, we still believe that our results are valuable, as this diagnostic strategy mirrors real-world clinical practice, where surgery is performed on a small percentage of patients. Finally, while glucose testing is highly effective for distinguishing between mucinous and non-mucinous pancreatic cystic lesions, it does not directly assess malignancy. Malignant potential is often associated with additional features, such as mural nodules or high-grade dysplasia, which can be identified using advanced imaging techniques like contrast-enhanced EUS, microvascular assessment, and elastography. These techniques, in combination with glucose testing, could improve overall diagnostic accuracy and risk stratification. Future studies should explore the integration of glucose measurement with these complementary tools to better evaluate malignancy.

## 5. Conclusions

Glucose fluid analysis by on-site glucometry of samples obtained via EUS–FNA, using a cut-off of 50 mg/dL, is a promising tool for differentiating between mucinous and non-mucinous PCLs. This method provides a rapid, cost-effective, and reproducible diagnostic approach with high diagnostic accuracy. Our results validated the glucose cut-off in a multicentric prospective cohort, thereby supporting the integration of on-site glucose measurement into standard diagnostic protocols for PCLs. 

## Figures and Tables

**Table 1:** Comparison of clinical and pancreatic cysts characteristics based on intracystic glucose levels.

Characteristics	*n*	Overall, *n* = 50 ^1^	Glucose < 50 mg/dL, *n* = 37 (74%) ^1^	Glucose ≥ 50 mg/dL, *n* = 13 (26%) ^1^	*p*-Value ^2^
**Age**	50	70 (65, 74)	71 (67, 74)	66 (64, 73)	0.4
**Diabetes mellitus**	50	9 (18%)	4 (30.8%)	5 (12.8%)	0.15
**Intracystic glucose (laboratory)**	22	53.8 (48.5)	10.3 (8.5)	97.3 (26.5)	<0.001
**Intracystic CEA**	29	41 (3, 286)	146 (8, 400)	3 (2, 17)	0.047
**Serum CA19.9**	9	22 (6, 27)	22 (10, 25)	47 (24, 70)	0.9
**Amylase**	14	108 (5, 361)	326 (116, 640)	5 (5, 5)	0.031
**Number of cysts**	50	1.00 (1.00, 2.00)	1.00 (1.00, 2.00)	1.00 (1.00, 1.00)	0.4
**Maximum diameter cyst (mm)**	50	22 (15, 36)	20 (13, 30)	35 (25, 55)	0.017
**Maximum diameter mural nodules (mm)**	6	7.5 (4.3, 10.0)	5.0 (4.5, 7.5)	10.0 (7.0, 17.5)	0.7
**Main pancreatic duct size (mm)**	50	2.50 (2.13, 3.00)	3.00 (2.50, 4.00)	2.50 (2.00, 2.50)	0.027
**Gender (Male)**	50	21 (42%)	16 (43%)	5 (38%)	0.8
**Region of pancreas involved by sampled cyst**	50				0.2
Tail		5 (10%)	5 (14%)	0 (0%)	
Body		15 (30%)	11 (30%)	4 (31%)	
Head		20 (40%)	14 (38%)	6 (46%)	
Tail and body		4 (8.0%)	2 (5.4%)	2 (15%)	
Body and head		5 (10%)	5 (14%)	0 (0%)	
Uncinate		1 (2.0%)	0 (0%)	1 (7.7%)	
**Cyst diagnosis**	50				<0.001
M-PCLs		33 (66%)	30 (81%)	3 (23%)	
NM-PCLs		17 (34%)	7 (19%)	10 (77%)	
**Cyst wall thickening**	50	11 (22%)	5 (14%)	6 (46%)	0.023
**Mural nodules inside cyst**	50	6 (12%)	3 (8.1%)	3 (23%)	0.2
**Vascular enhanced mural nodules**	50	5 (10%)	2 (5.4%)	3 (23%)	0.10
**Peripancreatic adenopathy**	50	3 (6.0%)	0 (0%)	3 (23%)	0.015
**FNA cytology**	50				0.2
Serous cyst		12 (24%)	5 (14%)	7 (54%)	
Mucinous cyst		3 (6.0%)	2 (5.4%)	1 (7.7%)	
High-grade dysplasia		6 (12%)	5 (14%)	1 (7.7%)	
Low-grade dysplasia		2 (4.0%)	2 (5.4%)	0 (0%)	
No neoplastic cells		23 (46%)	19 (51%)	4 (31%)	
Insufficient specimen		2 (4.0%)	2 (5.4%)	0 (0%)	
No clear pathologic diagnosis		2 (4.0%)	2 (5.4%)	0 (0%)	
**Histopathology on surgical specimen**	11				0.3
Mucinous cyst		1 (9.1%)	0 (0%)	1 (50%)	
High-grade dysplasia		5 (45%)	4 (44%)	1 (50%)	
Low-grade dysplasia		3 (27%)	3 (33%)	0 (0%)	
No neoplastic cells		2 (18%)	2 (22%)	0 (0%)	

^1^ Median (IQR); *n* (%); ^2^ Wilcoxon rank-sum test; Pearson’s chi-squared test; Fisher’s exact test.

**Table 2:** Comparison of clinical and pancreatic cysts characteristics of M-PCLs and NM-PCLs.

Characteristic	*n*	Overall, *n* = 50 ^1^	M-PCLs, *n* = 33 (66%) ^1^	NM-PCLs, *n* = 17 (34%) ^1^	*p*-Value ^2^
**Age**	50	70 (65, 74)	71 (67, 76)	65 (58, 73)	0.017
**Diabetes mellitus**	50	9 (18.0%)	6 (18.2%)	3 (17.6%)	0.9
**Intracystic Glucose (on-site)**	50	25 (18, 49)	22 (14, 29)	84 (29, 100)	<0.001
**Intracystic Glucose (laboratory)**	22	53.7 (48.5)	26.3 (32.1)	93.4 (40.5)	0.003
**Intracystic CEA**	29	41 (3, 286)	180 (17, 344)	3 (2, 56)	0.10
**Serum CA19.9**	9	22 (6, 27)	24 (18, 60)	4 (2, 5)	0.078
**Amylase**	14	108 (5, 361)	227 (108, 483)	5 (5, 189)	0.12
**Number of cysts**	50	1.00 (1.00, 2.00)	1.00 (1.00, 3.00)	1.00 (1.00, 1.00)	0.3
**Maximum diameter cyst (mm)**	50	22 (15, 36)	20 (15, 30)	30 (15, 55)	0.2
**Maximum diameter mural nodules (mm)**	6	7.5 (4.3, 10.0)	5.0 (4.5, 7.5)	10.0 (7.0, 17.5)	0.7
**Main pancreatic duct size (mm)**	50	2.50 (2.13, 3.00)	3.00 (2.50, 3.00)	2.50 (2.00, 2.50)	0.2
**Gender (Male)**	50	21 (42%)	15 (45%)	6 (35%)	0.5
**Region of pancreas involved by sampled cyst**	50				0.8
Tail		5 (10%)	4 (12%)	1 (5.9%)	
Body		15 (30%)	9 (27%)	6 (35%)	
Head		20 (40%)	13 (39%)	7 (41%)	
Tail and body		4 (8.0%)	3 (9.1%)	1 (5.9%)	
Body and head		5 (10%)	4 (12%)	1 (5.9%)	
Uncinate		1 (2.0%)	0 (0%)	1 (5.9%)	
**Cyst wall thickening**	50	11 (22%)	3 (9.1%)	8 (47%)	0.004
**Mural nodules inside cyst**	50	6 (12%)	3 (9.1%)	3 (18%)	0.4
**Vascular enhanced mural nodules**	50	5 (10%)	2 (6.1%)	3 (18%)	0.3
**Peripancreatic adenopathy**	50	3 (6.0%)	1 (3.0%)	2 (12%)	0.3
**Glucose < 50 mg/dL**	50	37 (74%)	30 (91%)	7 (41%)	<0.001
**FNA cytology**	50				<0.001
Serous cyst		12 (24%)	2 (6.1%)	10 (59%)	
Mucinous cyst		3 (6.0%)	3 (9.1%)	0 (0%)	
High-grade dysplasia		6 (12%)	6 (18%)	0 (0%)	
Low-grade dysplasia		2 (4.0%)	2 (6.1%)	0 (0%)	
No neoplastic cells		23 (46%)	17 (52%)	6 (35%)	
Insufficient specimen		2 (4.0%)	2 (6.1%)	0 (0%)	
No clear pathologic diagnosis		2 (4.0%)	1 (3.0%)	1 (5.9%)	
**Histopathology on surgical specimen**	11				0.3
Mucinous cyst		1 (9.1%)	1 (10%)	0 (0%)	
High-grade dysplasia		5 (45%)	5 (50%)	0 (0%)	
Low-grade dysplasia		3 (27%)	3 (30%)	0 (0%)	
No neoplastic cells		2 (18%)	1 (10%)	1 (100%)	

^1^ Median (IQR); *n* (%); ^2^ Wilcoxon rank-sum test; Pearson’s chi-squared test; Fisher’s exact test.

**Table 3:** Diagnostic accuracy of single cyst fluid biomarkers for a correct diagnosis of PCLs.

Marker	Sensitivity %	Specificity %	Accuracy %	AUC	Odds Ratio (OR)
Glucose < 50 mg/dL	93.2	76.5	89.0	0.74 (95% CI: 0.618 to 0.879)	14.29 (95% CI: 3.09 to 65.99)
CEA > 192 ng/mL	55.6	87.5	75.8	0.65 (95% CI: 0.487 to 0.817)	4.44 (95% CI: 0.74 to 26.68)

## Data Availability

The data supporting the reported results are not publicly available due to privacy and ethical restrictions.

## References

[1] Zerboni G., Signoretti M., Crippa S., Falconi M., Arcidiacono P.G., Capurso G. (2019). Systematic review and meta-analysis: Prevalence of incidentally detected pancreatic cystic lesions in asymptomatic individuals. Pancreatology.

[2] de Jong K., Nio C.Y., Hermans J.J., Dijkgraaf M.G., Gouma D.J., van Eijck C.H., van Heel E., Klass G., Fockens P., Bruno M.J. (2010). High prevalence of pancreatic cysts detected by screening magnetic resonance imaging examinations. Clin. Gastroenterol. Hepatol..

[3] Gonda T.A., Cahen D.L., Farrell J.J. (2024). Pancreatic Cysts. N. Engl. J. Med..

[4] Ohno E., Balduzzi A., Hijioka S., De Pastena M., Marchegiani G., Kato H., Takenaka M., Haba S., Salvia R. (2024). Association of high-risk stigmata and worrisome features with advanced neoplasia in intraductal papillary mucinous neoplasms (IPMN): A systematic review. Pancreatology.

[5] Mao K.Z., Ma C., Song B. (2024). Radiomics advances in the evaluation of pancreatic cystic neoplasms. Heliyon.

[6] European Study Group on Cystic Tumours of the Pancreas (2018). European evidence-based guidelines on pancreatic cystic neoplasms. Gut.

[7] Gardner T.B., Park W.G., Allen P.J. (2024). Diagnosis and Management of Pancreatic Cysts. Gastroenterology.

[8] Lisotti A., Napoleon B., Facciorusso A., Cominardi A., Crinò S.F., Brighi N., Gincul R., Kitano M., Yamashita Y., Marchegiani G. (2021). Contrast-enhanced EUS for the characterization of mural nodules within pancreatic cystic neoplasms: Systematic review and meta-analysis. Gastrointest. Endosc..

[9] Assifi M.M., Nguyen P.D., Agrawal N., Dedania N., Kennedy E.P., Sauter P.K., Prestipino A., Winter J.M., Yeo C.J., Lavu H. (2014). Non-neoplastic Epithelial Cysts of the Pancreas: A Rare, Benign Entity. J. Gastrointest. Surg..

[10] Bellocchi M.C.C., Manfrin E., Brillo A., Bernardoni L., Lisotti A., Fusaroli P., Parisi A., Sina S., Facciorusso A., Gabbrielli A. (2024). Rare pancreatic/peripancreatic cystic lesions can be accurately characterized by EUS with through-the-needle biopsy—A unique pictorial essay with clinical and histopathological correlations. Diagnostics.

[11] Gu A., Li J., Qiu S., Hao S., Yue Z.-Y., Zhai S., Li M.-Y., Liu Y. (2024). Pancreatic cancer environment: From patient-derived models to single-cell omics. Mol. Omics.

[12] Ohtsuka T., Castillo C.F.-D., Furukawa T., Hijioka S., Jang J.-Y., Lennon A.M., Miyasaka Y., Ohno E., Salvia R., Wolfgang C.L. (2024). International evidence-based Kyoto guidelines for the management of intraductal papillary mucinous neoplasm of the pancreas. Pancreatology.

[13] Sinha S.R., Mondal S., Jawed Akhtar M., Kumar Singh R., Prakash P. (2024). Evaluating Carcinoembryonic Antigen and Glucose Levels in Pancreatic Cyst Fluid for Mucinous Versus Non-mucinous Differentiation. Cureus.

[14] Williet N., Caillol F., Karsenti D., Abou-Ali E., Camus M., Belle A., Chaput U., Levy J., Ratone J.-P., Tournier Q. (2023). The level of glucose in pancreatic cyst fluid is more accurate than carcinoembryonic antigen to identify mucinous tumors: A French multicenter study. Endosc. Ultrasound..

[15] Gyimesi G., Keczer B., Rein P., Horváth M., Szűcs Á., Marjai T., Szijártó A., Hritz I. (2024). Diagnostic performance of intracystic carcinoembryonic antigen (CEA) versus glucose in differentiation of mucinous and non-mucinous pancreatic cysts. Pathol. Oncol. Res..

[16] Thornton G.D., McPhail M.J.W., Nayagam S., Hewitt M.J., Vlavianos P., Monahan K.J. (2013). Endoscopic ultrasound guided fine needle aspiration for the diagnosis of pancreatic cystic neoplasms: A meta-analysis. Pancreatology.

[17] Zikos T., Pham K., Bowen R., Chen A.M., Banerjee S., Friedland S., Dua M.M., A Norton J., A Poultsides G., Visser B.C. (2015). Cyst fluid glucose is rapidly feasible and accurate in diagnosing mucinous pancreatic cysts. Am. J. Gastroenterol..

[18] Ribaldone D.G., Bruno M., Gaia S., Cantamessa A., Bragoni A., Caropreso P., Sacco M., Fagoonee S., Saracco G.M., De Angelis C. (2020). Differential diagnosis of pancreatic cysts: A prospective study on the role of intra-cystic glucose concentration. Dig. Liver Dis..

[19] Iglesias-Garcia J., Lariño-Noia J., Abdulkader I., Domínguez-Muñoz J.E. (2014). Rapid on-site evaluation of endoscopic-ultrasound-guided fine-needle aspiration diagnosis of pancreatic masses. World J. Gastroenterol..

[20] Ribeiro T., Vilas-Boas F., Lopes S., Moutinho-Ribeiro P., Macedo G. (2023). Performance of Intracystic Glucose Measurement for the Characterization of Pancreatic Cystic Lesions. J. Gastrointest. Liver Dis..

[21] Noia J.L., Mejuto R., Oria I., De la Iglesia-García D., Villaverde A., Voces A., Pizzala J., Iglesias-García J., Urgiles D., Marcolongo M. (2022). Rapid diagnosis of mucinous cystic pancreatic lesions by on-site cyst fluid glucometry. Surg. Endosc..

[22] McCarty T.R., Garg R., Rustagi T. (2021). Pancreatic cyst fluid glucose in differentiating mucinous from nonmucinous pancreatic cysts: A systematic review and meta-analysis. Gastrointest. Endosc..

[23] Thiruvengadam N., Park W.G. (2015). Systematic review of pancreatic cyst fluid biomarkers: The path forward. Clin. Transl. Gastroenterol..

[24] Gorris M., Dijk F., Farina A., Halfwerk J.B., Hooijer G.K., Lekkerkerker S.J., Voermans R.P., Wielenga M.C., Besselink M.G., van Hooft J.E. (2023). Validation of combined carcinoembryonic antigen and glucose testing in pancreatic cyst fluid to differentiate mucinous from non-mucinous cysts. Surg. Endosc..

[25] Siddappa P.K., Park W.G. (2023). Pancreatic Cyst Fluid Analysis. Gastrointest. Endosc. Clin. N. Am..

[26] Oria I., Lariño-Noia J., Villaverde A., Pizzala J.E., Pasqua A., Urgiles D., Garcia D.D.L.I., Mejuto R., Iglesias-Garcia J., Mazza O.C. (2020). Sa1413 Cyst Fluid Glucose Obtained by EUS-FNA is Accurate for the Diagnosis of Mucinous Pancreatic Cysts. Experience from Two Terciary Care Centers. Gastrointest. Endosc..

[27] Carr R.A., Yip-Schneider M.T., Simpson R.E., Dolejs S., Schneider J.G., Wu H., Ceppa E.P., Park W., Schmidt C.M. (2018). Pancreatic cyst fluid glucose: Rapid, inexpensive, and accurate diagnosis of mucinous pancreatic cysts. Surgery.

[28] Rossi G., Petrone M.C., Tacelli M., Zaccari P., Crippa S., Belfiori G., Aleotti F., Locatelli M., Piemonti L., Doglioni C. (2024). Glucose and lactate levels are lower in EUS-aspirated cyst fluid of mucinous vs. non-mucinous pancreatic cystic lesions. Dig. Liver Dis..

[29] Faias S., Pereira L., Roque R., Chaves P., Torres J., Cravo M., Pereira A.D. (2020). Excellent Accuracy of Glucose Level in Cystic Fluid for Diagnosis of Pancreatic Mucinous Cysts. Dig. Dis. Sci..

[30] Park W.G., Wu M., Bowen R., Zheng M., Fitch W.L., Pai R.K., Wodziak D., Visser B.C., Poultsides G.A., Norton J.A. (2013). Metabolomic-derived novel cyst fluid biomarkers for pancreatic cysts: Glucose and kynurenine. Gastrointest. Endosc..

